# Division of Stricture without Subsequent Sounding

**Published:** 1884-04

**Authors:** J. McF. Gaston

**Affiliations:** Atlanta, Georgia


### DIVISION OF STRICTURE WITHOUT SUBSEQUENT SOUNDING.

BY J. MCF. GASTON, M. D., ATLANTA, GEORGIA.

Urethral surgery has advanced within the last decade from an art to the distinction of a science; and, yet, with all the improvements that have been introduced, it becomes a matter of serious consideration that results have not been secured which give entire satisfaction in the operation of urethrotomy.

While commendable progress has been made in the explorations of the canal of the urethra, and in the mode of detecting its obstructions, the steps adopted for the removal of the greater and less impediments to the flow of the urine, differ widely in the hands of the several operators, while the instruments most appropriate for the division of stricture are not precisely determined.

Specialists in this department are not agreed among themselves as to the safest and most efficient process of treatment; gradual dilitation, forcible and rapid dilatation, incision jointly with dilatation, incision followed by dilating measures, and incision simply and purely, being preferred for overcoming strictures by those having experience in these respective processes.

There is a difference of opinion among those who use cutting instruments, as to making the incision along the upper or lower wall of the canal, and the liability to grave consequences from the complete division of strictures that are deep-seated by internal or external operations.

Having noted the controversy in regard to these points, with an eye solely to arrive at the correct mode of proceeding, and having put into practice that which seemed most conducive to the end in view, I have to present some simple and plain facts to the consideration of those who, like myself, are called upon occasionally to operate for stricture of the urethra. It is not expected that the class of specialists, who are for the most part wedded to their individual mode of proceeding, will give any attention to the views of one based upon a much more limited experience, but the surgeon who includes these cases, with others, in his general operations, may be prepared to receive my suggestion as worthy to be adopted in his practice.

Ten years ago, I witnessed in the Misericordia hospital of Rio de Janeiro, an operation upon an almost impermeable stricture of the urethra, with the instrument of Maissoneuve, in the hands of Dr. V. Saboia, the professor of surgery in the Imperial Medical Academy. He passed the largest lamina belonging to this urethrotome, and afterwards a corresponding bougie was carried readily into the bladder. He then stated that no bougie, or other dilator, would be introduced into the urethra again until the subject came for inspection ten days afterwards; and further insisted that when a stricture is completely cut through, as he had done in this case, there was no indication for the use of any means of dilatation, while the introduction of the bougie, or catheter, was invariably a source of trouble. When the subject of this operation presented himself ten days subsequently, he made the declaration that no difficulty had attended the passage of urine, and that no instrument of any kind had been introduced since that used immediately after the incision of the urethra. Dr. Saboia, on this occasion, carried the same large bougie into the bladder, with like facility as at the outset; and after the lapse of another ten days without interference, he passed it again, illustrating most conclusively that the preservation of the tract of the urethra, did not in any way depend upon the subsequent use of means of dilatation. This man was now directed to report in future, should any further difficulty occur in the passage of urine, but otherwise the case was dismissed as cured.

A new light beamed in upon urethral surgery from this case; and having observed frequently, prior to this, untoward results from the passage of catheters after division of the urethra with the instrument of Civiale, I determined to adopt not only the instrument of Maissoneuve. but to refrain entirely from the use of all means of dilatation subsequently.

During the past ten years, a number of close strictures, and some of them located at the bulbo-membranous division of the urethra, have been divided with the urethrotome of Maissoneuve, leaving the cases for ten days, or two weeks, without interference, and then only using a large bougie a single time to verify the free opening of the canal. No case thus treated has failed to be completely relieved, without giving any of the disagreeable concomitants of rigors, fever, and local irritation which had formerly attended the introduction of bougies or catheters.

The most important feature of this operation is the entire division of the stricture in whatever division of the urethra it may exist; and when one of the smaller blades of the instrument has been used, it may occur that a bougie that glides easily through the other portion of the canal will encounter more or less difficulty in passing the partially divided stricture.. In such cases the larger lamina should be employed at once, so as to insure the complete division of the tissues implicated in the stricture, and the urethrometre should be applied subsequently, so that no doubt may remain in regard to the opening being equal to the caliber of the sound portion of the canal.

If any explanation is requisite for the full comprehension of the principle involved in non-interference after incision, it may suffice to state that the contractile tissue composing the stricture, being completely divided at one point, draws away from this line on both sides, leaving a gaping open space, so that no union can ensue if left to heal by granulation of this raw surface. The use of dilating tubes of any kind is calculated to irritate this exposed surface, and expose the case to various troubles, with which all are unfortunately too familiar to require any enumeration in this place.

One other condition for the favorable result of this operation is, that the incision be made in the lower wall of the urethra, and not above, as has been practiced by a distinguished specialist and his followers in this country. The strictured band is divided more certainly by an incision below, and with less danger of hemorrhage or urinary infiltration than if made in the upper wall along the corpora cavernosa.

The distortion of the penis, which has sometimes occurred from circumscribed effusion and consequent inflammation in the tissues of this organ from incisions in the upper wall of the spongy division of the canal, should serve as a caution against operating in this manner.

All the good results of dividing strictures may be secured by making the incision in the lower wall of the urethra, without risking the consequences of a deep incision above, and I cannot understand, with all the lights before me, why the division should be so often made by specialists along the superior wall of the canal. The reasoning presented by its advocates is not satisfactory, and the occasional unfavorable results of the practice do not recommend it to our confidence.

In presenting this result of a somewhat limited personal observation in urethral surgery, it may be proper to say that I have continued this practice, after having all the appliances of Dr. Fessenden N. Otis explained to me by himself, and after reading, with great care and much interest, his able production on "Stricture of the Male Urethra." In the hands of that skillful operator, there is no doubt but that good results are secured with his instruments, yet in the fair test given them by Mr. Berkeley Hill, in London, the fruits of his experience did not encourage the continued employment of this process.

While Dr. Otis ordinarily resorts to the subsequent "separation of the wound throughout its extent, by the easy passage of a full-sized steel sound daily or every other day until the healing is complete;" yet he recognizes the fact that trouble may ensue from this procedure. "In my experience," he remarks, 'operations confined to the pendulous urethra are, as a rule, *never followed by constitutional disturbance*, even when six or seven strictures are divided at the same sitting. But to insure this result no instrument, not even a sound for exploratory purposes, should be passed into the bladder during or immediately subsequent to the operation."

Thus, he allows, that the cure of stricture may be effected without any sounding after the incision, which is made so as to insure the normal caliber to the canal; and it is evident that the constricting band being completely divided, the gaping margins require no mechanical separation by a sound, bougie, or other means of dilatation.

The urethrometre and bulbous sounds, as improved by Dr. Otis, are essential to a proper acquaintance with the location and extent of the strictures that may require operation, and by a proper preliminary examination the lamina of Maissoneuve's urethrotome may be adjusted according to the requirements of the case.

Should it be desirable to employ some other form of the urethrotome than Maissoneuve's, the instrument of Dr. Kinloch, cutting from behind forward, offers some advantages over that of Dr. Otis, and with its filiform appendage may be adapted to narrow strictures as well as to those of large caliber.

